# Physiological responses and cycle characteristics during double-poling versus diagonal-stride roller-skiing in junior cross-country skiers

**DOI:** 10.1007/s00421-021-04689-2

**Published:** 2021-04-24

**Authors:** Erik P. Andersson, Irina Hämberg, Paulo Cesar Do Nascimento Salvador, Kerry McGawley

**Affiliations:** 1 Swedish Winter Sports Research Centre, Department of Health Sciences, Mid Sweden University, Östersund, Sweden; 2 School of Sport Sciences, Faculty of Health Sciences, UiT The Arctic University of Norway, Tromsø, Norway; 3 Physical Effort Laboratory, Sports Center, Federal University of Santa Catarina, Florianopolis, Brazil

**Keywords:** Cross-country skiing, Cycle length, Cycle rate, Energy cost, Gross efficiency, Oxygen pulse, Oxygen uptake kinetics, Peak oxygen uptake, Sex differences, Winter sports

## Abstract

**Purpose:**

This study aimed to compare physiological factors and cycle characteristics during cross-country (XC) roller-skiing at matched inclines and speeds using the double-poling (DP) and diagonal-stride (DS) sub-techniques in junior female and male XC skiers.

**Methods:**

Twenty-three well-trained junior XC skiers (11 women, 12 men; age 18.2 ± 1.2 yr.) completed two treadmill roller-skiing tests in a randomized order using either DP or DS. The exercise protocols were identical and included a 5 min warm-up, 4 × 5 min submaximal stages, and an incremental test to exhaustion, all performed at a 5° incline.

**Results:**

No significant three-way interactions were observed between sex, submaximal exercise intensity, and sub-technique. For the pooled sample, higher values were observed for DP versus DS during submaximal exercise for the mean oxygen uptake kinetics response time (33%), energy cost (18%), heart rate (HR) (9%), blood lactate concentration (5.1 versus 2.1 mmol·L^−1^), rating of perceived exertion (12%), and cycle rate (25%), while cycle length was lower (19%) (all *P* < 0.001). During the time-to-exhaustion (TTE) test, peak oxygen uptake ($\dot{V}$O_2_peak), peak HR, and peak oxygen pulse were 8%, 2%, and 6% lower, respectively, for DP than DS, with a 29% shorter TTE during DP (pooled data, all *P* < 0.001).

**Conclusion:**

In well-trained junior XC skiers, DP was found to exert a greater physiological load than DS during uphill XC roller-skiing at submaximal intensities. During the TTE test, both female and male athletes were able to ski for longer and reached markedly higher $\dot{V}$O_2_peak values when using DS compared to DP.

## Introduction

Cross-country (XC) skiing involves the use of many different sub-techniques within classic and skate skiing, with double-poling (DP) and diagonal-stride (DS) being two of the most frequently used sub-techniques in classic skiing (Solli et al. 2018). Performance in XC skiing is characterized by high energetic demands (Andersson et al. 2019; Gløersen et al. 2019; Karlsson et al. 2018) and, as in other endurance sports, is related to maximal oxygen uptake, oxygen uptake at the lactate threshold ($\dot{V}$O_2_-LT), anaerobic capacity, and the energy cost of skiing (Hoffman and Clifford 1992; Losnegard 2019). In addition, these physiological measures are specific to the sub-technique employed. In a recent review by Losnegard (2019), peak oxygen uptake ($\dot{V}$O_2_peak) was reported to be ~ 12% lower in XC skiers using DP compared to DS, independent of sex. This is due to the lower relative contribution of the leg muscles during DP (Björklund et al. 2015). By contrast, relative $\dot{V}$O_2_ (as a % of $\dot{V}$O_2_peak) and blood lactate concentration ([La]) were found to be significantly higher during DP compared to DS when performing submaximal roller-skiing at a 4° gradient using matched incremental treadmill speeds (Hoffman et al. 1994; Mittelstadt et al. 1995). For gross energy cost (EC), a crossover point has been demonstrated in male XC skiers, with DS becoming more economical than DP at inclines steeper than ~ 3° (Andersson et al. 2017).

In a laboratory environment, Pellegrini et al. (2013) showed that well-trained men almost never self-selected DP at gradients steeper than 4°, and all chose DS when the gradient reached 6°. Ettema et al. (2017) concluded that incline, rather than speed, was the factor that led to shifts in classic sub-technique choice in senior male XC skiers. Despite these findings, and the fact that DS has traditionally been used during uphill skiing, the use of DP in steep terrains has become more common in recent years (Pellegrini et al. 2018). In addition, elite long-distance XC ski races are now mainly won by both sexes using exclusively DP (Sagelv et al. 2018; Stöggl et al. 2020). These changes have been attributed to physiological and biomechanical developments in modern XC skiers, particularly related to the upper body (Stöggl and Holmberg 2011; Welde et al. 2017), as well as improvements in skiing equipment and track preparation (Sandbakk and Holmberg 2014). Improvements in functional characteristics of the skis and poles, together with more well-prepared ski tracks, have decreased the net EC of skiing and have contributed to increased XC skiing speeds (Formenti et al. 2005). In addition, modern XC skiers utilize a DP technique that is characterized by an active flexion–extension pattern in the elbow, hip, knee, and ankle joints, which represents a complex movement pattern that incorporates more than just upper body work (Holmberg et al. 2005). Moreover, $\dot{V}$O_2_peak in DP relative to DS has gradually increased over the last 50 years (Stöggl et al. 2019), which is probably related to the increased relative usage of DP in competitions over the last decade (Stöggl and Holmberg 2011; Stöggl et al. 2019, 2020). Moreover, skiers choosing to use DP exclusively in competition may benefit from reduced sliding friction between skis and snow, due to the lack of grip wax, which is otherwise required in DS (Stöggl et al. 2019).

To prevent traditional sub-techniques from becoming extinct in classic XC skiing, the International Ski Federation (FIS) has introduced technical zones on certain uphill sections of competition tracks where DP is forbidden (Pellegrini et al. 2018). This rule change, together with demanding, hilly course profiles, means that DS continues to be an important classical skiing sub-technique over the traditional distances. From an athlete-development perspective, it is important to understand the differing demands of DP and DS in junior XC skiers. This information may in turn be used to generate and disseminate knowledge to athletes and coaches. However, previous studies have exclusively described and compared physiological and biomechanical responses to DP and DS in senior XC skiers. Given the differences in muscle mass in junior versus senior athletes (Aerenhouts et al. 2012) and the relative differences in upper to lower body muscle mass distribution and strength between women and men (Heyward et al. 1986; Janssen et al. 2000; Levine et al. 1984), this leaves an important gap in the scientific literature.

The present study aimed to compare physiological responses and cycle characteristics during submaximal and maximal DP and DS roller-skiing using a standardized 5° uphill protocol in a group of junior XC skiers. A further aim was to investigate differences in responses between the sexes. It was hypothesized that uphill DP would be more physically demanding than DS during submaximal exercise, reflected in a higher $\dot{V}$O_2_, [La], heart rate (HR), rating of perceived exertion (RPE), and EC. During maximal exercise, a longer time-to-exhaustion (TTE), higher $\dot{V}$O_2_peak, and higher peak HR (HR_peak_) were expected during DS compared to DP. Based on previous research (Sandbakk et al. 2014), it was hypothesized that the differences in physiological responses and cycle characteristics would be greater between the two sub-techniques for the women compared to the men.

## Methods

### Participants

Twenty-three junior XC skiers (11 women and 12 men) were recruited and participated voluntarily, and the participant characteristics are shown in Table 1. Inclusion criteria required that all participants were studying at a specialist ski high school, were born between 1998–2001, were currently competing in the highest national cross-country skiing competition, and had trained at least 350 h (for the 17–18 years old) or 450 h (for the 19–20 years old) in the preceding year. Any XC skier not attending a specialist ski high school, not volunteering to participate in the study, or not meeting all of the inclusion criteria were naturally excluded from the sample. The participants received verbal and written information relating to the study, including the possible risks and advantages of participating. All participants were required to provide written informed consent and those under the age of 18 yr. were required to provide additional informed consent from a parent or guardian. All participants were able to terminate participation in the study at any time without providing an explanation. The study was pre-approved by the Regional Ethics Committee in Umeå, Sweden (2018/154-31).

**Table 1:** Participant characteristics (mean ± SD)

	All (*n* = 23)	Women (*n* = 11)	Men (*n* = 12)
Age (yr.)	18.2 ± 1.2	18.2 ± 1.4	18.2 ± 1.0
Height (cm)	175.5 ± 7.8	169.8 ± 5.0*	180.8 ± 6.0
Body mass (kg)	69.1 ± 8.6	63.5 ± 6.9*	74.3 ± 6.6
Training hours previous year	551.3 ± 83.9^a^	535.3 ± 79.8^b^	564.6 ± 88.4

*Significantly different from the men (*P* < 0.001)

^a^*n* = 22

^b^*n* = 10 (due to an incomplete training log)

### Study overview

**Fig. 1:**
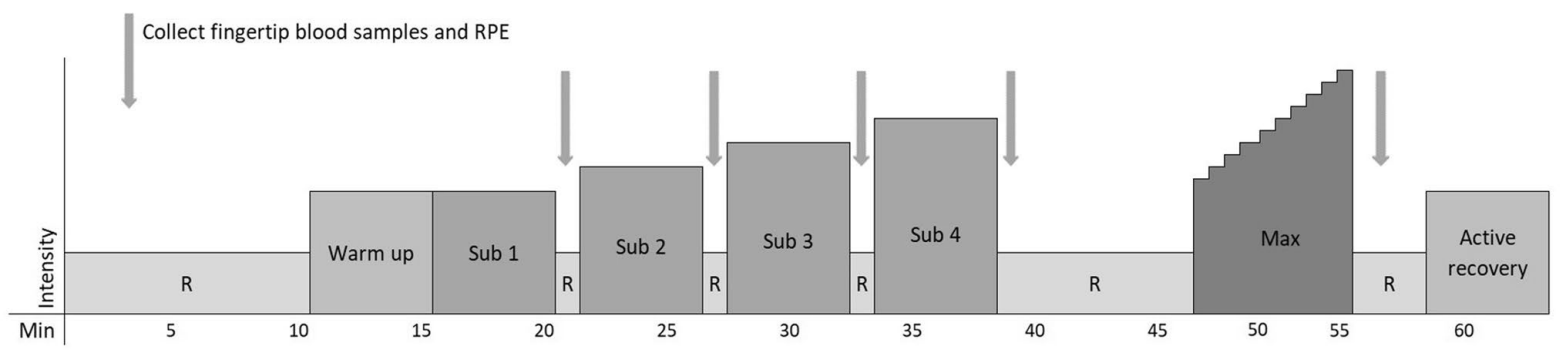
A schematic of the test protocol used during both the double-poling and diagonal-stride trials. *R* passive rest

Participants arrived at the laboratory in a rested state having refrained from ingesting caffeine or alcohol, or using nicotine, for the previous 24 h. The maximum training load prescribed in the 24 h prior to testing was up to 2 h of low-intensity exercise. Participants were asked to replicate the same training and food intake before the second trial as before the first. The test protocol (Fig. 1) was identical for both the DP and DS trials and the order of completing each sub-technique was randomized and counter-balanced. Height and body mass were measured on arrival at the laboratory, after which the participants rested in a seated position and filled out training background and health forms. After 8 min of seated rest, a resting blood sample was collected for the analysis of [La], and resting HR was measured.

Four different submaximal test protocols were developed and each participant was assigned to one of the four protocols depending on their fitness level, which was assessed from previous incremental tests of maximal oxygen uptake or 3000 m running tests, and/or skiing performance during the previous winter season. A familiarization session was completed no more than 6 weeks before performing the first experimental trial, with athletes roller-skiing on a treadmill for 2 min at each of their proposed submaximal intensities using both the DP and DS sub-techniques. During the experimental trials, initial speeds for the four submaximal protocols were either 5, 6, 7, or 8 km·h^−1^, and the treadmill speed was increased by 1 km·h^−1^ per stage for all protocols, with each stage lasting 5 min. All protocols used a fixed uphill gradient of 5° (~ 8.75%), which was chosen to enable “proper” skiing using both DP and DS at the prescribed speeds (rather than “DS walking” at the slower speeds, for example). Speeds for the first submaximal stage within each protocol were also selected to enable participants to work within the moderate-intensity exercise domain (i.e., below the lactate threshold). An initial warm-up was completed for 5 min at the same intensity as for the first submaximal stage. The incremental TTE test protocol started after 8 min of passive rest at the same workload as the second submaximal stage, and speed was increased by 0.4 km·h^−1^ and 0.6 km·h^−1^ alternately every 30 s, resulting in a speed increase of 1 km·h^−1^ each minute. This method was used due to the control system of the treadmill only allowing speed increments of 0.2 km·h^−1^.

### Equipment and measurements

All tests were performed on a motor-driven treadmill developed for roller-skiing (Rodby Innovation AB, Vänge, Sweden). For safety reasons, the treadmill was equipped with two emergency brakes, one situated next to the test leader and one connected to a safety harness that was attached around the skier’s waist. All of the athletes used the same pair of Swix C2 roller-skis (Sterner specialfabrik AB, Dala-Järna, Sweden) equipped with an NNN binding system (Rottefella AS, Klokkarstua, Norway). To avoid differences in rolling resistance, the skis were pre-warmed in a heating box for at least 30 min at a temperature of 34 °C. The coefficient of rolling resistance was 0.022 and the total mass of the roller-skis was 2.3 kg. The athletes used their own ski boots and poles with a weight of ~ 1.1 kg. Pole length was standardized for all tests and was 82 ± 1% (and ≤ 83%) of body height for all participants, which is consistent with the FIS pole-length rule. Prior to exercise, the pole basket was exchanged for carbide tips designed for treadmill roller-skiing.

The height and body mass of the participants were measured before the tests using an electronic scale (Seca 764, Hamburg, Germany). Respiratory variables were analyzed using a portable breath-by-breath system, with a total weight of 1.2 kg (MetaMax 3B, Cortex, Leipzig, Germany). The system was calibrated according to the recommendations by first calibrating the gas sensor with fresh ambient air and then calibrating against a reference gas with fixed concentrations of 15% O_2_ and 5% CO_2_ (UN 1950 Aerosols, Cortex Biophysik GmbH, Leipzig, Germany). The turbine’s flow volume was calibrated with a 3 L syringe (M9474-C, Medikro Oy, Kuopio, Finland). HR was monitored continuously during all tests using a standard chest strap and wristwatch (Polar V800, Polar Electro, Kempele, Finland). $\dot{V}$O_2_ and HR were analyzed for the last 60 s of each submaximal stage and $\dot{V}$O_2_peak was calculated as the highest 30 s moving average during the incremental TTE test. For the calculation of peak oxygen pulse, HR over the same time frame as for the $\dot{V}$O_2_peak was used. Blood samples were collected from the fingertip in 20 µL capillary tubes and were subsequently analyzed for [La] (Biosen S-line, EKF Diagnostics, Cardiff, UK). RPE was reported using the 6–20-point Borg Scale (Borg 1982) after each submaximal stage, the TTE test, and the subsequent recovery period.

The power output for submaximal roller-skiing was calculated as the power to overcome the rolling resistance and to elevate the total system mass (*m*_sys_: the sum of body mass and equipment mass) against gravity:

1 $$ {\text{Power}}\;{\text{output}}\;[W]\; = \;vm_{{{\text{sys}}}} \;(g\;\sin \;(\alpha )\; + \;\mu_{R} g\;\cos \;(\alpha )), $$

where *g* is gravitational acceleration,* v* is the treadmill speed [m∙s^−1^], *µ*_*R*_ is the rolling resistance coefficient, and *α* is the treadmill incline (Andersson and McGawley 2018). Energy expenditure was calculated from the $\dot{V}$O_2_ (L∙min^−1^) and respiratory exchange ratio (*RER*) according to Weir (1949) and then converted to a metabolic rate (*MR*):

2 $$ MR\;[W]\;\; = \;\frac{{4184\;(\dot{V}{\text{O}}_{2} \;(1.1RER\; + 3.9))}}{60} $$

using average $\dot{V}$O_2_ and *RER* values from the final minute of each stage of the submaximal exercise protocol (an *RER* value of 1.00 was used in the cases during submaximal DP where *RER* > 1.00). Gross *EC* relative to *m*_sys_ was expressed as:

3 $$ EC\;\left[ {{\text{J}} \;. \;{\text{Kg}}^{ - 1} \; . \;{\text{m}}^{ - 1} } \right]\; = \;\frac{MR}{{vm_{{{\text{sys}}}} }}. $$

Gross efficiency (GE) was calculated as the ratio between power output and *MR* and expressed as a percentage.

The $\dot{V}$O_2_ kinetics mean response time (*MRT*, defined as the time to reach 63% of the complete $\dot{V}$O_2_ response) was analyzed over the first 10 min of submaximal exercise (i.e., the warm-up and the first submaximal stage). This 600-s time window demonstrated the lowest standard error of estimate (SEE) for all transitions (*MRT* SEE during DP = 1.91 ± 0.42 s; *MRT* SEE during DS = 1.82 ± 0.37 s). Breath-by-breath data were processed and analyzed as described previously (do Nascimento Salvador et al. 2018), with each condition initially examined to exclude any outlying values (caused by sighs, swallowing, and coughs, for example). The breath-by-breath data were then linearly interpolated to 1 s intervals and reduced to a 5 s stationary average to decrease the influence of signal error and to improve parameter estimation (Rossiter 2011). Non-linear regression techniques were used to fit the data after the onset of a fundamental phase with an exponential function (OriginPro 8, OriginLab, Northampton, MA, USA). An iterative process ensured that the sum of squared errors was minimized. The model was constrained by the baseline $\dot{V}$O_2_ to aid in the identification of the key parameters according to the following equation:

4 $$ \dot{V}{\text{O}}_{2} (t)\; = \;{\text{baseline}}\;\dot{V}{\text{O}}_{2} \; + \;Ax\;\left[ {1\; - \;{\text{e}}^{{\left( \frac{t}{MRT} \right)}} } \right], $$

where $\dot{V}$O_2_ (*t*) represents the value of $\dot{V}$O_2_ at a given time, *t*, baseline $\dot{V}$O_2_ is the average $\dot{V}$O_2_ value calculated over the final 60 s of rest, *A* is the asymptotic amplitude for the exponential term describing changes in $\dot{V}$O_2_ from baseline to its asymptote, and *MRT* is the early phase time constant that represents the time to 63% of the total $\dot{V}$O_2_ response. A mono-exponential function starting at the onset of the exercise was considered the most appropriate approach for characterizing the overall *MRT*.

A Go-Pro camera (GoPro Hero 1, GoPro Inc., San Mateo, CA, USA) was used to record all tests for the subsequent analysis of cycle rate and cycle length. A skiing cycle was defined as the moment from the start of the pole plant until the same pole made contact with the treadmill belt again and cycles were counted during the last 60 s of each submaximal stage. For the calculation of cycle rate, the number of full cycles completed within the last 60 s of the submaximal stage was divided by the exact time taken to complete those cycles. The cycle length was then calculated by dividing speed by cycle rate.

### Statistical analyses

The Statistical Package for the Social Sciences (SPSS 25, IMB Corp, Armonk, NY, USA) was used for the statistical analyses and the level of significance was set at *P* < 0.05. Data are presented as mean ± standard deviation (SD), except in the case of RPE, where data are presented as the median and interquartile range (IQR). Data were checked for normality by visual inspection of the histograms and the assumption of sphericity was tested using Mauchly’s test, where in the case of violated sphericity (i.e., epsilon < 0.75), the degrees of freedom were corrected using the Greenhouse–Geisser correction. A three-way ANOVA was used to investigate the effects of exercise intensity × sub-technique × sex during submaximal exercise. In the absence of any effect of sex, a two-way ANOVA was subsequently used to analyze the effects of exercise intensity × sub-technique. Separate two-way ANOVA tests were used to investigate the effects of sex × sub-technique for *MRT* and during the incremental TTE test, with post hoc paired *t* tests used to compare variables for DP versus DS. For RPE during the submaximal stages, the main effect of sub-technique was analyzed by comparing the DP and DS median values using a Wilcoxon *z* test, and the main effect of intensity was analyzed by comparing the four medians from the four submaximal stages for DP and DS. For RPE during the incremental TTE test, the main effect of sub-technique was also analyzed using a Wilcoxon *z* test, while the main effect of sex was analyzed with a Mann–Whitney *U* test based on median data for DP and DS.

## Results

### Submaximal exercise

**Fig. 2:**
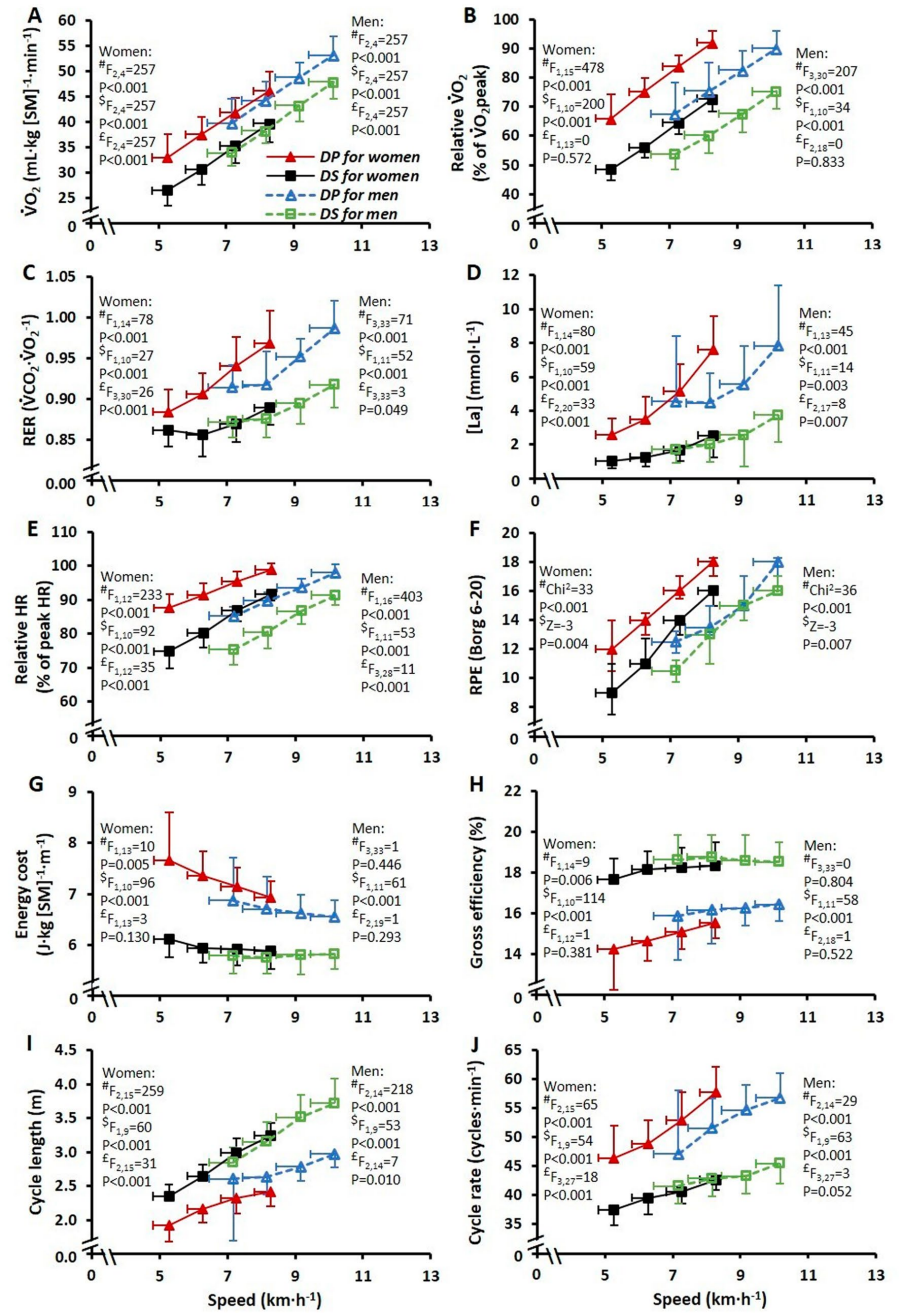
Physiological, perceptual, and cycle-characteristic responses during uphill (5º) roller-skiing using the double-poling (DP; triangular markers) and diagonal-stride (DS; square markers) sub-techniques at four submaximal speeds for males (blue and green markers) and females (red and black markers). The values are presented as mean ± SD except for RPE, which is presented as median and interquartile range. *F* and *P* statistics are presented for: ^#^effect of speed; ^$^effect of sub-technique; ^£^interaction effect between speed and sub-technique. Nonparametric alternatives were used for rating of perceived exertion (RPE). $$\dot{V}$$*O*_*2*_ oxygen uptake, *RER* respiratory exchange ratio, *[La]* blood lactate concentration, *HR* heart rate, *RPE* rating of perceived exertion

**Fig. 3:**
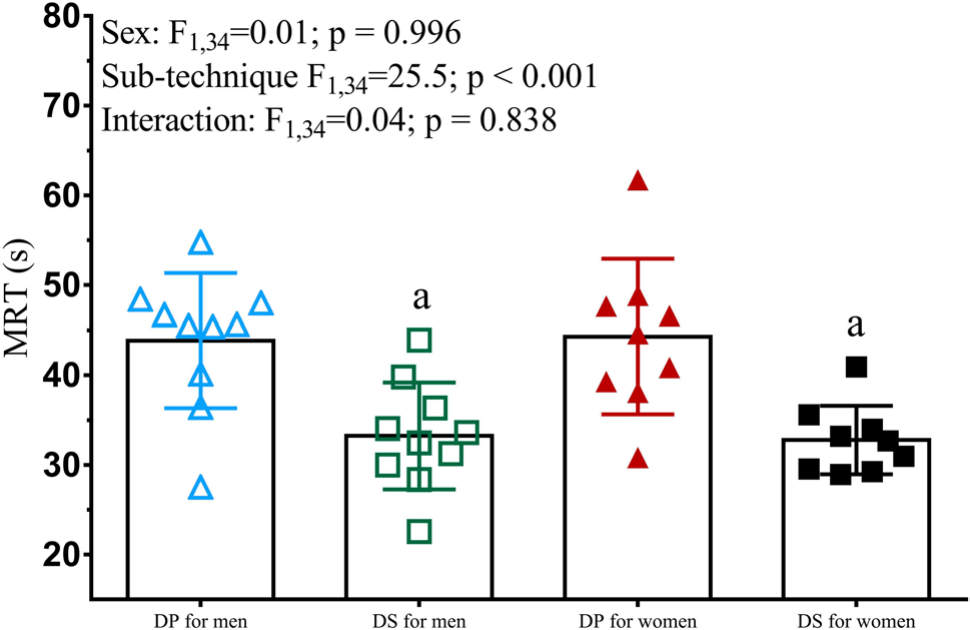
Mean ± SD pulmonary $\dot{V}$O_2_ mean response time (*MRT*) during double-poling (DP; triangular markers) and diagonal-stride (DS; square markers) sub-techniques for males (blue and green markers) and females (red and black markers)

Results from the three-way ANOVA showed no interaction effects between exercise intensity, sub-technique, and sex for any of the measured variables during submaximal exercise. Therefore, two-way ANOVAs were used to analyze the effects of sub-technique and exercise intensity for all 23 skiers (Table 2). The $\dot{V}$O_2_, *RER*, [La], HR, RPE, EC, and cycle rate were all significantly higher for DP versus DS, while GE and cycle length were significantly lower (all *P* < 0.001). In addition, there were significant effects of submaximal intensity for all variables (all *P* ≤ 0.002), with increases from the first to the last submaximal stage in all cases except for EC, which decreased significantly in DP with higher exercise intensity (*P* = 0.008). The $\dot{V}$O_2_, *RER*, [La], HR, RPE, EC, GE, cycle length, and cycle rate responses are displayed in Fig. 2 for the women and men separately. The $\dot{V}$O_2_ kinetics *MRT* data (*n* = 18; 9 women and 9 men) are displayed in Fig. 3, where it can be seen that DP induced a significantly longer *MRT* compared to DS.

**Table 2:** Responses to the four submaximal stages (Sub 1–4) of uphill (5°) treadmill roller-skiing using diagonal-stride (DS) and double-poling (DP) for both sexes combined (*n* = 23)

		Sub 1	Sub 2	Sub 3	Sub 4	*P*
Speed (km·h^−1^)	–	6.3 ± 1.1	7.3 ± 1.1	8.3 ± 1.1	9.3 ± 1.1	–
Power output (W)	–	140 ± 37	162 ± 39	184 ± 41	206 ± 43	–
$\dot{{\text{V}}}$O_2_ (mL·kg m_sys_^−1^·min^−1^)	DS	30.3 ± 4.6	34.6 ± 4.8	39.5 ± 5.2	44.0 ± 5.4	< 0.001
	DP	36.5 ± 5.7	41.0 ± 4.9	45.5 ± 4.5	49.7 ± 5.1	< 0.001
						0.729
RER	DS	0.87 ± 0.02	0.87 ± 0.03	0.88 ± 0.03	0.90 ± 0.03	< 0.001
	DP	0.90 ± 0.03	0.91 ± 0.03	0.95 ± 0.03	0.98 ± 0.03	< 0.001
						< 0.001
[La] (mmol·L^−1^)	DS	1.4 ± 0.7	1.6 ± 0.9	2.1 ± 1.4	3.2 ± 1.5	< 0.001
	DP	3.1 ± 1.3	4.0 ± 1.6	5.4 ± 2.0	7.7 ± 2.9	< 0.001
						< 0.001
HR (beats·min^−1^)	DS	150 ± 13	160 ± 12	173 ± 11	183 ± 10	< 0.001
	DP	169 ± 9	177 ± 9	185 ± 9	192 ± 9	< 0.001
						< 0.001
RPE (Borg 6–20)	DS	10 (7–14)	12.5 (10–15)	14 (12–16)	17 (14–18)	< 0.001
	DS	12 (9–15)	14 (11–16)	16 (13–17)	18 (16–19)	< 0.001
						–
EC (J·kg m_sys_^−1^·m^−1^)	DS	5.9 ± 0.4	5.8 ± 0.3	5.9 ± 0.3	5.9 ± 0.3	< 0.001
	DP	7.2 ± 1.0	7.0 ± 0.65	6.9 ± 0.45	6.7 ± 0.4	0.002
						0.046
GE (%)	DS	18.2 ± 1.2	18.5 ± 1.0	18.4 ± 1.1	18.5 ± 1.0	< 0.001
	DP	15.1 ± 2.2	15.5 ± 1.6	15.7 ± 1.0	16.0 ± 0.9	< 0.001
						< 0.001
^a^CL (m)	DS	2.6 ± 0.3	2.9 ± 0.4	3.2 ± 0.4	3.5 ± 0.4	< 0.001
	DP	2.2 ± 0.4	2.4 ± 0.3	2.6 ± 0.3	2.7 ± 0.4	< 0.001
						< 0.001
^a^CR (cycles·min^−1^)	DS	39.6 ± 3.3	41.3 ± 3.1	42.0 ± 2.9	43.8 ± 2.8	< 0.001
	DP	47. 9 ± 5.3	50.1 ± 4.8	53.5 ± 4.5	56.7 ± 3.9	< 0.001
						< 0.001

Values are presented as mean ± SD except for rating of perceived exertion (RPE), which is presented as median and interquartile range. *P* values are presented for each of the variables for sub-technique, intensity, and interaction effects, respectively, except for RPE where non-parametric statistics are presented for sub-technique and sex

$\dot{V}$*O*_*2*_ oxygen uptake, *m*_*sys*_ system mass (i.e., the sum of body mass and equipment mass), *RER* respiratory exchange ratio, *[La]* blood lactate concentration, *HR* heart rate, *EC* energy cost, *GE* gross efficiency, *CL* cycle length, *CR* cycle rate

^a^*n* = 20 for CL and CR (due to lost data as a result of technical issues with the GoPro files)

### Maximal TTE test

The performance, physiological, and perceptual responses to the maximal TTE test are displayed in Table 3, along with results from the paired *t* tests comparing responses for all 23 skiers and the two-way ANOVA tests showing sex, sub-technique, and interaction effects. There were significant effects of sub-technique on performance (i.e., TTE), $\dot{V}$O_2_peak and HR_peak_, with significantly higher values attained for DS compared to DP (*P* < 0.001). By contrast, there were no significant differences between sub-techniques for [La] or RPE responses. Sex effects were identified for $\dot{V}$O_2_peak and HR_peak_, with higher values attained by the men in both cases (*P* < 0.001 and *P* = 0.036, respectively). The only interaction effect between sub-technique and sex was identified for [La], where the men elicited a higher [La] following DP and women elicited a higher [La] following DS (*P* = 0.029).

**Table 3:** Responses to the incremental time-to-exhaustion test using diagonal-stride (DS) and double-poling (DP) treadmill roller-skiing at 5°

		All	*T* test	Women	Men	ANOVA
		(*n* = 23)	*P*	(*n* = 11)	(*n* = 12)	*P*
Time to exhaustion (s)	DS	451 ± 38	< 0.001	456 ± 23	447 ± 48	< 0.001
	DP	320 ± 47		307 ± 30	332 ± 57	0.548
						0.130
$\dot{V}$O_2_peak (mL·kg BM^− 1^·min^− 1^)	DS	^a^63.2 ± 7.1	< 0.001	58.6 ± 3.3	^a^67.9 ± 6.9	< 0.001
	DP	^a^58.4 ± 7.1		53.7 ± 4.5	^a^63.1 ± 6.1	< 0.001
						0.986
[La] (mmol·L^−1^)	DS	10.9 ± 2.5	0.518	11.1 ± 2.6	10.7 ± 2.5	0.420
	DP	10.5 ± 3.5		9.4 ± 2.5	11.5 ± 3.4	0.475
						0.029
HR_peak_ (beats·min^−1^)	DS	200 ± 9	< 0.001	195 ± 8	204 ± 8	< 0.001
	DP	196 ± 9		192 ± 9	199 ± 7	0.036
						0.350
Peak O_2_ pulse	DS	31.7 ± 3.2	< 0.001	30.0 ± 2.0	33.4 ± 3.5	< 0.001
([mLO_2_·beat^−1^·kg BM^−1^] × 100)	DP	29.8 ± 3.3		28.0 ± 3.0	31.7 ± 2.6	< 0.001
						0.421
RPE (Borg 6–20)	DS	19 (17–20)	0.820	19 (17–20)	19.5 (19–20)	0.082
	DP	19 (17–20)		19 (17–20	19 (17–20)	0.107

Values are presented as mean ± SD except for rating of perceived exertion (RPE), which is presented as median and interquartile range. *T* test *P* statistics are presented for sub-technique comparisons for all participants combined (*n* = 23). ANOVA *P* statistics are presented for sub-technique, sex, and interaction effects, respectively, except for RPE where non-parametric statistics are presented for sub-technique and sex

$\dot{V}$*O*_*2*_*peak* peak oxygen uptake, *BM* body mass, *[La]* blood lactate concentration, *HR* heart rate, *peak O*_*2*_* pulse* peak oxygen pulse

^a^*n* minus 1, due to a technical issue with the metabolic cart on one occasion

## Discussion

The current study aimed to compare physiological responses and cycle characteristics during classic XC skiing using the DP and DS sub-techniques on a 5° uphill incline, in a group of female and male junior XC skiers. In support of the hypothesis, DP was found to be more physically demanding than DS during standardized submaximal exercise, demonstrated by significantly greater $\dot{V}$O_2_, [La], HR, RPE, and EC responses. Moreover, *MRT* was significantly longer in DP compared with DS, whereas relative $\dot{V}$O_2_ (as a % of $\dot{V}$O_2_peak), *RER*, and cycle rate were significantly higher and GE and cycle length were significantly reduced. In further support of the hypothesis, TTE was longer and $\dot{V}$O_2_peak and HR_peak_ were higher for DS compared to DP during the maximal test. Contrary to the hypothesis, however, there was no evidence for greater differences in physiological responses and cycle characteristics between the two sub-techniques for the women compared to the men.

The hypothesis that differences in physiological responses and cycle characteristics would be greater between DP and DS for the women compared to the men was based on previous studies, demonstrating that women have a relatively lower amount of upper versus lower body muscle mass (Janssen et al. 2000) and that women exhibit larger differences in upper compared to lower body strength compared to men (Heyward et al. 1986; Levine et al. 1984; Miller et al. 1993). Moreover, findings from Sandbakk et al. (2014) revealed that the sex-specific difference in XC roller-skiing performance was larger for DP (at a 5% incline) than DS (at a 12% incline) during a TTE test. The lack of a significant two-way interaction effect between sub-technique and sex on TTE performance in the current study was in contrast to our hypothesis and the previous findings observed by Sandbakk et al. (2014). However, this might be related to the different study design and/or age group employed in the current study. Also for submaximal exercise, and contrary to our hypothesis, no significant three-way interactions were observed between sex, submaximal exercise intensity, and sub-technique in the current study. As such, the submaximal data were analyzed for the pooled sample (Table 2) and were displayed visually for the two sexes individually for illustrative purposes only (Fig. 2).

The majority of the outcomes during submaximal exercise in the present study, presented in Fig. 2 and Table 2, are supported by previous research that has compared DP and DS among senior (i.e., collegiate and national-level) female and male XC skiers using a treadmill incline of 4° (7.1%) and standardized graded treadmill speeds (Hoffman et al. 1994; Mittelstadt et al. 1995). Specifically, these authors found relative $\dot{V}$O_2_ (as a % of $\dot{V}$O_2_peak), *RER*, [La], and RPE to be greater during DP compared to DS, which is similar to the findings of the current study. The difference in the distribution of arm- and leg-muscle involvement during DP and DS is likely to affect the metabolic responses, as well as the associated feelings of perceived exertion. For example, van Hall et al. (2003) showed considerable increases in [La] (from < 3.0 to > 7.5 mmol∙L^−1^) and *RER* (from ~ 0.93 to 1.00) when transferring from DS (i.e., involving substantial contributions from both the arms and legs) to DP (i.e., involving greater contributions from the arms) at an exercise intensity of 72–76% of maximal oxygen uptake. They also reported a far greater lactate release in the arms during DP compared to DS, which relative to muscle mass could not be matched by lactate uptake and resulted in greater lactate accumulation during DP.

Contrary to the current findings, Hoffman et al. (1994) observed no significant differences between sub-techniques for submaximal $\dot{V}$O_2_ (in mL·kg^−1^·min^− 1^) or HR. This might, to some extent, be due to the lower treadmill gradient compared to the current study (i.e., a 4° versus 5° slope). For example, $\dot{V}$O_2_ is substantially lower during DP versus DS on flat terrain (Hoffman and Clifford 1990; Hoffman et al. 1994; Saibene et al. 1989), indicating that DP becomes progressively more metabolically demanding, relative to DS, as the slope becomes steeper. However, the findings from these previous studies should be interpreted with caution, since they were conducted approximately 3 decades ago and the DP sub-technique has evolved considerably since the 1990s, with increased relative importance of DP to race performance, and therefore greater prioritization of the sub-technique in training (Sandbakk and Holmberg 2014; Stöggl et al. 2019, 2020). In a more recent study, Sagelv et al. (2018) showed senior male elite long-distance and national-level all-round (i.e., traditional) XC skiers to elicit an ~ 9% higher submaximal $\dot{V}$O_2_ for uphill (6°) DP compared to DS at 8 km·h^−1^, but a similar submaximal $\dot{V}$O_2_ between sub-techniques at 10 km·h^−1^. This is in contrast to the results presented in the current study, where the submaximal $\dot{V}$O_2_ was higher for DP than DS at all speeds, from 6.3 ± 1.1 km·h^−1^ to 9.3 ± 1.1 km·h^−1^. The divergent findings are likely to be explained, at least to some extent, by the higher age (~ 26 years) and training volume (713 h·year^−1^) of the senior male skiers in comparison to the mixed-sex group of junior skiers in the current study. The precise ways in which $\dot{V}$O_2_, HR, EC/GE, and other physiological and biomechanical variables are altered with DP and DS at varying gradients among junior compared to senior, and female compared to male XC skiers remains to be investigated. This information could be used to help inform training practices and racing strategies across athlete groups.

In the current study, submaximal EC was ~ 18% higher for DP compared to DS. This is consistent with previous results showing DS to be more economical than DP at inclines steeper than ~ 3° (Andersson et al. 2017; Pellegrini et al. 2013). In addition, DP became more economical at progressively higher speeds in the current study, with EC decreasing and GE increasing over the four submaximal stages. By contrast, EC and GE were relatively constant during DS. This is in agreement with previous findings (Andersson et al. 2017, 2020; Sagelv et al. 2018), and the differences in EC and GE between the two sub-techniques are likely to be related to several biomechanical and physiological factors. During DS, propulsion is generated by both the arms and legs in a diagonal fashion, while propulsion during DP is generated solely through the poling action (Nilsson et al. 2004). Previous studies have shown that the relative propulsive phase over a movement cycle (i.e., the duty cycle) is substantially shorter for DP than DS (Lindinger et al. 2009a; Stöggl and Holmberg 2016). This is likely to result in larger speed fluctuations over the movement cycle for DP compared to DS while roller-skiing at a 5° incline, which could partly explain the higher EC for DP than DS in the current study (Millet et al. 1998). Moreover, the substantially greater involvement of the upper body relative to the lower body during DP compared to DS could result in different efficiencies at a muscular level, due to the different motor units involved and/or contractile characteristics of the recruited muscle fibers (Calbet et al. 2005; He et al. 2000; Suzuki 1979).

This is the first study, to our knowledge, that has analyzed $\dot{V}$O_2_ kinetics during XC skiing for DP versus DS, with results revealing a significantly faster *MRT* in DS compared to DP by 11.0 ± 8.5 s. A fast *MRT* can be advantageous, as it increases the contribution of aerobic metabolism to exercise and reduces the need for anaerobic energy production at the start of exercise (Hajoglou et al. 2005), thereby sparing finite anaerobic resources for later high-intensity bouts or spurts, which would be beneficial to maximal performance (Burnley and Jones 2007). Previous studies have shown that the $\dot{V}$O_2_ kinetics *MRT* is exercise-mode specific and might be longer for upper compared to lower body exercise (Casaburi et al. 1992; Koppo et al. 2002). The current study supports this suggestion, with a longer response time for the more upper body-dominant DP sub-technique (Björklund et al. 2015; Stöggl et al. 2013). Furthermore, different task-specific muscle recruitment patterns and contraction types lead to different $\dot{V}$O_2_ kinetics *MRT*, independent of sex (do Nascimento Salvador et al. 2018; Pringle et al. 2002).

Several physiological and biomechanical factors are probably related to the 35.6% longer $\dot{V}$O_2_ kinetics *MRT* in DP compared to DS. For instance, oxygen extraction has been reported to be substantially lower for DP than DS at similar relative (Björklund et al. 2015) and absolute (Calbet et al. 2005) exercise intensities. The lower oxygen extraction in DP versus DS has been attributed to a more heterogeneous blood flow for upper versus lower body exercise in general (Kalliokoski et al. 2004), an irregular blood flow during the high muscle activation ski-specific poling phase (i.e., a mechanical hindrance of blood flow) (Stöggl et al. 2013), a smaller diffusing area, higher blood pressure, a shorter mean transit time, and a longer diffusing distance (Björklund et al. 2015; Calbet et al. 2004, 2005; Volianitis and Secher 2002). Most of the aforementioned factors are likely to be related to the substantially slower $\dot{V}$O_2_ kinetics for DP versus DS observed in the current study. It is also possible that the different characteristics of the muscle groups involved in DP compared to DS (Calbet et al. 2005; Ørtenblad et al. 2018) contributed to these findings.

It was hypothesized that during the incremental TTE test, the relative difference between DP and DS would be larger for the female than the male skiers, as observed by a significant two-way interaction effect. Although this interaction effect did not reach significance (*P* = 0.130), the mean TTE was relatively shorter during DP compared to DS for the women (33% shorter) compared to the men (26% shorter). A potential explanation for the larger difference in the women can be seen in Fig. 2g, where EC tended to be higher for the female skiers at similar submaximal DP speeds compared to the male skiers. This finding is accompanied by a shorter cycle length (Fig. 2i) and higher cycle rate (Fig. 2j) for the women. The fact that this was considerably more pronounced in DP than DS indicates that women may benefit more than men from upper body-specific training that is designed for increasing cycle length in DP. The only interaction effect between sub-technique and sex was identified for [La] (see Table 3), where the men elicited a higher [La] following DP and the women following DS (*P* = 0.029). This might be due to the relatively lower muscle mass in the upper compared to the lower body in women (Gallagher and Heymsfield 1998; Janssen et al. 2000), which could affect the total magnitude of muscle lactate production during DP versus DS.

In a review by Losnegard (2019), DP was shown to elicit ~ 12% lower (range 5–18%) $\dot{V}$O_2_peak values compared to DS or running, and this was independent of sex. In the current study, the female and male skiers recorded $\dot{V}$O_2_peak values that were 7.1% and 8.4% lower, respectively, in DP compared to DS. A recent study has reported an even smaller difference in senior male skiers, with a 4% lower value in DP versus DS (Andersson et al. 2020). The greater emphasis on increasing upper body strength and endurance in modern XC skiers, compared to 2–5 decades ago, appears to have reduced this difference in $\dot{V}$O_2_peak values between DP and DS (Stöggl et al. 2019). Therefore, it is likely that the junior athletes in the current study will be able to reduce the difference in $\dot{V}$O_2_peak between DP and DS even further with additional years of training. While the mean $\dot{V}$O_2_peak for the whole group was 7.7% lower in DP versus DS, mean HR_peak_ was only 2.0% lower in DP. More important than HR_peak_ might be the peak oxygen pulse, which was 5.8% lower in DP. This could be explained by the lower oxygen extraction during DP compared to DS, which has been demonstrated in several previous studies (Björklund et al. 2015; Calbet et al. 2005; Stöggl et al. 2013), and/or a lower maximal stroke volume (Calbet et al. 2004).

Despite the substantially higher demands of DP compared to DS at a 5° uphill incline observed in the current study, XC skiers have started exclusively using DP during long-distance races (Sagelv et al. 2018; Stöggl et al. 2020). The advantage of DP is the improved glide in the absence of grip wax, which may compensate for the higher physiological loading at steeper inclines during DP. This is especially relevant over longer duration and relatively flatter races that are characteristic of long-distance XC ski competitions. In a recent study, Stöggl et al. (2019) systematically analyzed the influence of using DP exclusively on a FIS-approved, 5.3 km track versus using grip-waxed skis also allowing for the utilization of all the other grip-wax dependent classic sub-techniques (including DS). Findings showed the senior male elite skiers to be considerably faster when using DP exclusively, the junior men to be equally fast in both conditions, and the women (juniors and seniors) to be considerably slower when using DP only. These results highlight that age, sex, and performance level, in combination with the course profile, need to be considered when making decisions about whether to use DP exclusively in a race situation. Based on the considerably larger difference in EC/GE observed between uphill DP and DS in the current study, compared to the results presented by Sagelv et al. (2018) on senior elite long-distance skiers, the younger junior skiers in the current study could likely benefit from more upper-body-specific endurance and strength training for improving the EC/GE of DP.

When analyzing EC/GE, the submaximal metabolic rate should be at a steady state with *RER* < 1.00 and no distinct upward drift in [La]. Since DP was found to be considerably more physiologically demanding than DS during the submaximal exercise in the current study, six athletes reached *RER* values that were ~ 1.025 during the final submaximal stage when using DP. In addition, the [La] responses during the submaximal stages using DP were relatively high. Therefore, it is likely that the true metabolic rates during the submaximal exercise using DP would have been higher, at least for the upper stages, than the calculated values. Based on this, the true EC values for submaximal DP would probably have been slightly higher (and GE lower) than those reported. Also, the differences in EC/GE observed for treadmill roller-skiing with DP and DS may not be precisely the same as for XC skiing on snow, since DS roller-skiing enables static friction that is several times greater than for DS on snow, which allows a high propulsive leg force generation without the ski slipping (Ainegren et al. 2013). For the comparison of the incremental TTE performances between DP and DS, the participants were likely more fatigued after the submaximal protocol performed with DP, which may have magnified the differences in TTE performances between sub-techniques. Moreover, the total anaerobic energy provision during the TTE test was likely to be more limited in DP than DS, indicated by the markedly higher baseline [La] prior to the DP TTE test. Despite the aforementioned methodological limitations, the current study provides important new information about the differences in physiological responses and cycle characteristics during DP and DS when using identical protocols conducted on the same uphill incline in junior female and male XC skiers.

Due to the extensive amount of existing research on DP treadmill roller-skiing conducted in a laboratory (Holmberg et al. 2006, 2005; Lindinger et al. 2009b; Stöggl and Holmberg 2011, 2016), more research is needed that combines treadmill roller-skiing tests with outdoor performance tests on snow, similar to the previous study by Stöggl et al. (2019). This is particularly relevant when comparing the effects of classic XC skiing performance while using DP exclusively (i.e., without using grip-waxed skis) versus the use of grip-waxed skis that also allows for the use of all the other classic sub-techniques. Based on the increased importance of DP performance to the overall results in modern classic XC skiing races (Stöggl and Holmberg 2011; Stöggl et al. 2019, 2020), detailed training studies that quantify both the physiological and biomechanical changes, and their relationships to performance, would be of high practical value to athletes and coaches.

In conclusion, the current study reveals that well-trained junior XC skiers exert a substantially greater physiological load during uphill XC roller-skiing at submaximal intensities while employing the DP sub-technique in comparison to DS. The $\dot{V}$O_2_ kinetics *MRT* was substantially longer for DP than DS. During the incremental TTE test, athletes were able to ski for longer and reached markedly higher (~ 8%) $\dot{V}$O_2_peak values during incremental exercise to exhaustion when using DS compared to DP. In contrast to the hypothesis, there were no significant interaction effects of sex on the responses to submaximal or maximal exercise when comparing the two sub-techniques.

## Data Availability

Data are available on request.

## Abbreviations

ANOVA: Analysis of variance
DP: Double poling
DS: Diagonal stride
EC: Energy cost
Eq.: Equation
GE: Gross efficiency
HR: Heart rate
HR_peak_: Peak heart rate
IQR: Interquartile range
[La]: Blood lactate concentration
MR: Metabolic rate
MRT: Mean response time
RER: Respiratory exchange ratio
RPE: Rating of perceived exertion
SD: Standard deviation
m_sys_: System mass
TTE: Time to exhaustion
$$\dot{V}{\text{O}}_{2}$$: Oxygen uptake
$\dot{V}{\text{O}}_{2}$peak: Peak oxygen uptake
$\dot{V}{\text{O}}_{2}$-LT: Oxygen uptake at the lactate threshold
XC: Cross-country
